# Mammalian Herbivores Alter the Population Growth and Spatial Establishment of an Early-Establishing Grassland Species

**DOI:** 10.1371/journal.pone.0147715

**Published:** 2016-02-05

**Authors:** Lauren L. Sullivan, Brent J. Danielson, W. Stanley Harpole

**Affiliations:** 1 Department of Ecology Evolution and Behavior, University of Minnesota, St. Paul, Minnesota, 55108, United States of America; 2 Department of Ecology, Evolution and Organismal Biology, Iowa State University, Ames, Iowa, 50011, United States of America; 3 Department of Physiological Diversity, Helmholtz Center for Environmental Research–UFZ, Permoserstr. 15, 04318 Leipzig, Germany; 4 German Centre for Integrative Biodiversity Research (iDiv) Halle-Jena-Leipzig, Deutscher Platz 5e, D-04103 Leipzig, Germany; 5 Institute of Biology, Martin Luther University Halle-Wittenberg, Am Kirchtor 1, 06108 Halle (Saale), Germany; Helmholtz Centre for Environmental Research (UFZ), GERMANY

## Abstract

Plant-herbivore interactions influence the establishment context of plant species, as herbivores alter the community context in which individual species establish, and the spatial relationship between individuals and their source population as plants invade. This relationship can be described using an establishment kernel, which takes into account movement through seed dispersal, and subsequent establishment of adults. Mammalian herbivores are hypothesized to influence plant population growth and establishment through a combination of consumption of seeds and seedlings, and movement of seeds. While the movement abilities of plants are well known, we have very few empirical mechanistic tests of how biotic factors like mammalian herbivores influence this spread potential. As herbivores of all sizes are abundant on the landscape, we asked the question, how do mammalian herbivores influence the population growth, spatial establishment, and the community establishment context of an early-recruiting native prairie legume, *Chamaecrista fasciculata*? We planted *C*. *fasciculata* in source populations within a four-acre tallgrass prairie restoration in plots with and without herbivores, and monitored its establishment with respect to distance from the source populations. We found that herbivores decreased population growth, and decreased the mean and range establishment distance. Additionally, *C*. *fasciculata* established more often without herbivores, and when surrounded by weedy, annual species. Our results provide insight into how the interactions between plants and herbivores can alter the spatial dynamics of developing plant communities, which is vital for colonization and range spread with fragmentation and climate change. Mammalian herbivores have the potential to both slow rates of establishment, but also determine the types of plant communities that surround invading species. Therefore, it is essential to consider the herbivore community when attempting to restore functioning plant communities.

## Introduction

Plant movement across the landscape is influenced by factors that affect both the numbers of seeds, or propagules, and the distance these propagules move [1]. Seed dispersal is the template for recruitment, as a dispersal kernel captures the net probability distribution of offspring density as a function of the numbers of propagules and the distance they move from their source [2,3]. In contrast, an establishment kernel, often termed an effective dispersal kernel, is the joint probability of the gain in individuals through dispersal, and the loss of individuals through mortality [4,5]. Plant movement ability might therefore increase with greater numbers of offspring produced, if offspring disperse farther, or if establishment rates increase. Biotic factors such as herbivores influence plant establishment through a variety of processes that could have opposing effects on the establishment kernel itself [6–8]. For example, herbivores might decrease seed number through consumption, which would impact population growth, but simultaneously increase dispersal distance through zoochory [9,10]. Thus, the net effect of herbivory on plant establishment kernels might not be easily predicted. Quantifying the establishment kernel integrates over multiple demographic processes that include dispersal, establishment, and survival, and provides a means to quantitatively compare the effects of important factors such as herbivory on plant population movement. However, the shapes of establishment kernels are relatively unknown in temperate grassland systems, especially when considering how biotic factors like herbivores alter them.

Herbivores influence the shape of establishment kernels in grassland plants in two ways; either indirectly by moving seeds and affecting the dispersal kernel, or directly by consuming seeds and seedlings once they have dispersed by affecting establishment and survival [4]. Large animals like mammals and birds have the potential to disperse seeds (i.e., zoochory) farther on the landscape relative to non-animal mediated dispersal [11] and foraging behavior such as seed-caching [12]. However, herbivores can also decrease the distance of wind-mediated dispersal by altering plant traits that affect dispersal. For example, decreasing the height of seed-releasing individuals through consumption could have negative effects on seed movement, as height of seed release is positively correlated with dispersal distance [13], or consumption of pre-dispersed seeds decreases the overall number of potential dispersers. In contrast, herbivores can impact a species’ establishment kernel directly by influencing the dispersed population through consumption of pre-reproductive individuals, and post-dispersed seeds [14,15]. Consumption of individual seeds by granivores or insect seed predation, and seedlings or adults by insect or mammalian herbivores, will decrease the population growth. However foraging in a pattern, (e.g., via density-dependent foraging), would shift the establishment kernel by moving the largest proportion of established individuals away from the parent plant, where density is highest [16–18].

**Fig 1:**
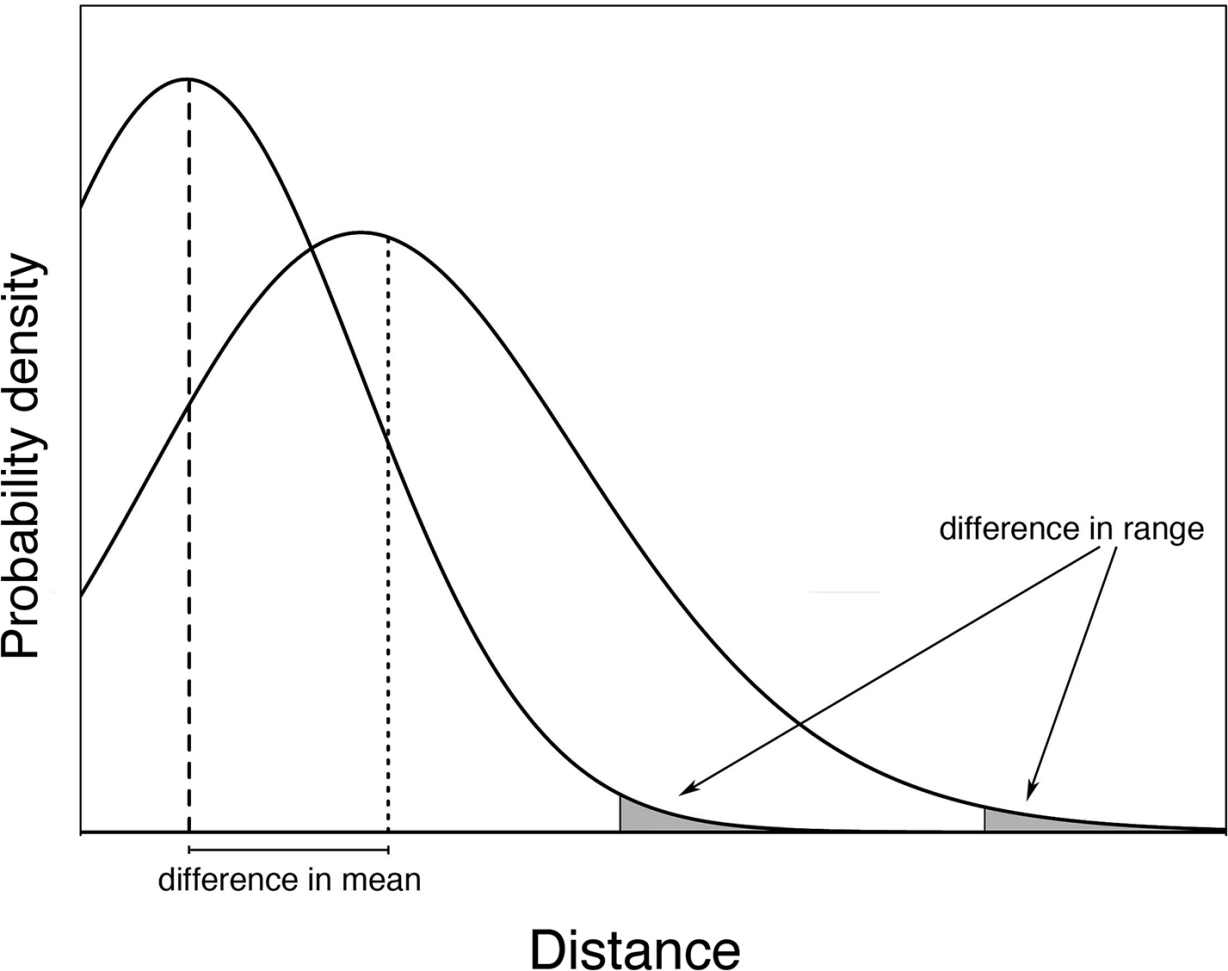
Conceptual curves describing how establishment kernel parameters can be used to show change in spatial processes. Here, two kernels are represented, which were created under similar conditions with the exception of the factor of interest (e.g.: herbivory). The entire black curve minus the gray area represent the body of the kernel, while the gray area depicts the tail of the kernel. The body of the kernel can be described by the mean distance, which is represented by the hatched lines. The tail, or range, of the distribution can be measured as the minimum distance travelled by the farthest 1% of individuals. Differences in these parameters can be used to determine how the factor of interest influences the spatial establishment of a target species.

Parameters from establishment kernels can be estimated and compared to determine the influences of herbivory on local spatial establishment of individuals. Specifically, different kernels can be compared by quantifying two aspects of the distributions: the body and the tail (Fig 1). The body of the distribution, which comprises the shape of the majority of probability mass, has direct application to spatial population growth and large-scale establishment [19,20]. Differences in the total number of individuals in the body of the kernel determines how herbivory affects overall population growth. The kernel mean describes the body, and determines the spatial dimension of central tendency of seed rain and subsequent establishment. Establishment kernels can also be compared by looking at the tail of the distribution, which determines the long distance dispersal (LDD) of the population. LDD is often described as the minimum distance travelled by the farthest 1% of individuals. In contrast to the body of the distribution, the tail is important for population expansion [21], and a key factor in recolonization over long distances [20,22].

We used a large-scale field experiment in a grassland restoration context to test the hypothesis that mammalian herbivores will impact plant population growth and establishment kernel parameters depending on their impacts on, and interactions with, the plant community. While there are many forms of herbivory that could influence establishment kernels, we focus here on mammalian herbivores specifically, as they are easier to experimentally exclude on the scale relevant to dispersal and establishment. Hereafter we will refer to them simply as “herbivores”. While the outcomes of herbivore influence are predicted to both increase and decrease establishment distance depending on the types of interactions (e.g., decrease with consumption, increase with apparent competition), the goal of this study was to demonstrate the impacts of herbivores on plant species invading from a source in an establishing grassland. This work is an exciting new step for movement ecology, as we empirically address mechanisms related to how the biotic community influences the movement ability of grassland species. Here, we used the native, early-recruiting prairie legume, *Chamaechrista fasciculata* (Michx.) as our study species. Using spatial data on the distance of established individuals from source populations into novel areas, as well as the plant community around each established stem, we asked the questions: do mammalian herbivores influence the shape of the establishment kernel of *C*. *fasciculata* populations? Additionally, if herbivores influence the establishment kernel of *C*. *fasciculata*, they may also affect the plant community in which this focal species establishes. Therefore, we also ask, do herbivores influence the competitive community in which *C*. *fasciculata* establishes? We show that herbivores exert negative pressure on *C*. *fasciculata* for both population growth and movement, which implicates their role in decreasing C. *fasciculata* invasion in newly-established grassland systems.

## Materials and Methods

### Study species

*Chamaecrista fasciculata* (Michx.), Partridge Pea (Fabaceae), is an early establishing annual legume common in grasslands and a variety of disturbed habitats across the eastern two thirds of the United States [23]. Plants are erect, ~0.5–1.5m tall, and produce indeterminate racemes of yellow, mostly outcrossing flowers [24], that are predominately bee pollinated [25]. Linear-oblong pods contain quadrate seeds that are elastically dehiscent, and thus disperse explosively. Plants flower from mid July to September, and fruit through October. We chose *C*. *fasciculata* as our study species because it is common in grassland restorations, and its life history necessitates quick establishment and ample dispersal. Adult plants are consumed by herbivores, and seeds are cached (Sullivan, pers. obs.). We based herbivory observations on plants being cut cleanly, and finding no evidence of the cut parts in the vicinity (i.e.: indicating consumption, not simply cutting). We could not distinguish which herbivores were cutting plants, simply that occurred. Adult plants are also visited by generalist insect herbivores including orthopterans, lepidopterans, and weevils, whose effect on seed production is significantly reduced by ant visitation to the extra-floral nectaries on *C*. *fasciculata*. [26,27]. As these insect herbivores are difficult to exclude, and significantly mediated by ants, we focus on mammalian herbivores as the prime herbivores influencing the movement of *C*. *fasciculata*.

### Site layout and preparation

We collected data from a tallgrass prairie restoration experiment in Ames, Iowa. This site was part of Iowa State University’s Research Farms, and was leased to the authors by funds provided by the Department of Ecology, Evolution and Organismal Biology. We seeded all areas of the restoration with 519 seeds/m^2^ with a ratio of 2/3 forbs and 1/3 grass seed by weight, in March 2012 after two rounds of tillage the previous fall. This seeding rate and density matched approximately those used in local, Iowa prairie restorations (Loren Lown, William Johnson, Dave Williams, Doug Sheeley, and Jon Judson; pers. comm.). The experiment contained eight experimental plots (32x32m^2^), each with a central 19.2m diameter circular core area. During seeding, we added *C*. *fasciculata* seeds to the seed mix for the central cores only. This ensured the creation of source populations of *C*. *fasciculata* surrounded by large, uncolonized areas.

We installed fencing around the outside of four plots to decrease herbivore abundance. The fences mainly excluded voles (*Microtus ochrogaster* (Wagner), and *M*. *pennsylvanicus* (Ord)) and white-tailed deer (*Odocoileus virginianus* (Boddart)), both of which have large effects on Midwestern grasslands [28]. To exclude voles, we buried hardware cloth 30 cm below ground, which extended 50 cm above ground [29]. This small fence also discouraged other burrowing animals from entering plots. We mowed one meter buffers on either side of this fence to further discourage voles from climbing into exclusion plots [29]. Periodically, we removed voles from fenced plots to maintain a low density of animals within the fences. To do this, we used Sherman Traps for 3 consecutive nights, and relocated all trapped animals. When necessary, animals were sacrificed using cervical separation. Our methods were reviewed and approved by the Iowa State University Institutional Animal Care and Use Committee (ISU IACUC permit 5-12-7373-W). We excluded deer using an offset electric fence [30]. We observed one herbivore-exclusion plot to have very high rates of herbivory due to leaky fences, while one herbivore plot had very low rates of herbivory, likely because it was bordered by two roads. We removed these plots from analysis after initial statistical tests showed they did not change trend directions, only increased variance.

### Data collection

To measure herbivore influence on population-level establishment of *C*. *fasciculata*, we counted all stems in 4 transects, 10 x 1.8m in size from each plot in the fall of 2013 (one year post sowing). Transects began at the edge of the core, and extending outward into the area not seeded with *C*. *fasciculata*. Therefore, distance zero for the establishment kernels started directly adjacent to *C*. *fasciculata* plantings. Thus these kernels represented population-level movement. We spatially located all counted stems, and defined establishment distance as the distance from the outer edge of the core to an established individual found within a transect. While transects were directional, and nested within each plot, due to low establishment in some herbivore transects (e.g.: 1–5 individuals) we could not interpret the effects of transect direction on total establishment. Therefore, one summed establishment kernel was created for each plot by combining distance data from all transects. The identity of transects with low establishment was not consistent across direction.

### Establishment kernel

To determine if herbivores had an effect on the overall establishment of *C*. *fasciculata*, we compared population growth between fencing treatments. We determined within-plot population growth to be the total number of individuals in the body of the non-normalized establishment kernel. To determine if herbivores influenced the shape of the establishment kernel, we constructed establishment kernels from the distance data, and extracted replicated population-level mean and range parameters from these distributions and compared the parameters across herbivore treatments.

### Competitive environment

To determine if competition with the plant community had additional influences on establishment, we measured the local competitive environment around each *C*. *fasciculata* stem. We quantified aerial cover in the 0.25 m^2^ surrounding each stem based on categories that included cover of planted native prairie species, cover of non-planted weedy species, and cover of bare ground. Because the canopy of these three types could overlap, these three percentages did not necessarily sum to 100%. By comparing the changes in cover of competing species between fenced and unfenced plots, we could also determine how herbivores mediated the effects of interspecific competition on *C*. *fasciculata* establishment.

### Statistical analysis

We ran our statistics using R v3.2.1, an open-source statistical computing language [31]. All data and R scripts to run all analysis and create all figures are found in the Supporting Information (S1 Code, S1 Data).

### Establishment kernel

Determining the influence of herbivores on the shape of the establishment kernel required that we describe the best functional form of the data before we could extract and compare meaningful parameters. To do this, we used maximum likelihood estimation to fit various distributions to our data. We considered distributions that are commonly used to describe dispersal and establishment [32]. Using the bblme package [33], we compared the normal (N), negative exponential (NE), negative binomial (NB), and Poisson (P) probability density functions to our data and selected the best-fit model using AIC. Of these, the NB distribution fit the establishment data best (NB (ΔAIC:0.0); NE (ΔAIC:28.7); N (ΔAIC:427.2); P (ΔAIC:3778.6); S1 Fig). We then estimated and compared parameters from the establishment kernels using a Bayesian approach.

We modeled the establishment distance (y) as a linear function of herbivore treatment using a Poisson-gamma mixture model [34]. This derivation of the NB distribution is common in ecology [32], and is useful for linear models as it allows for the direct parameterization of the kernel mean μ [35], a parameter of interest. Here, we modeled the likelihood as a Poisson distribution that is the product of two parameters (i.e., the mean μ, and dispersion ρ), where

$${\text{y}}_{\text{ijk}}∼\text{Poisson}(\mu \;*\;\rho ),$$ (1)

for individual i, in plot j and herbivore treatment k. We assume ρ to be equal across all treatments, and is distributed as

ρ∼Gamma(α,α). (2)

This formulation of the NB produces a distribution of μ's per plot, which we assume come from an overall distribution of μ per treatment, and is modeled as

$$\text{ln}(\mu_{\text{k}})=\beta_{0_{\text{j}}}+\beta_{1_{\text{j}}}\;*\;{\text{trt}}_{\text{k}}.$$ (3)

We used non-informative priors for all parameters. We modeled our slope $\beta_{0_{\text{i}}}$, and intercept $\beta_{1_{\text{i}}}$ parameters as N(δ, τ), with δ∼N(0,0.0001), and τ∼Gamma(0.0001, 0.0001). The parameter ln(α)∼Exp(0, 0.0001). To determine how herbivores influenced the mean of the establishment kernel μ_k_, we modeled plots independently and used simulated $\beta_{0_{\text{j}}}$ and $\beta_{1_{\text{j}}}$ values to calculate the estimated μ_k_'s per herbivore treatment from the NB model. We ran all Bayesian analyses using JAGS and the rjags package [36]. We allowed our data to converge using 5 chains, thinned parameters to eliminate autocorrelation, and removed all simulated parameters prior to convergence. We then used our μ_k_ and ρ parameters to simulate values of dispersal distance based on plots within treatments. This allowed us to capture the variation in our data. Using this set of simulated distances, we quantified the distance travelled by the farthest 1% of individuals, and compared these tail distances between treatments.

### Competitive environment

To determine if local competition with the plant community influenced the establishment of *C*. *fasciculata*, the number of stems were binned by cover of each of three types: percentage of cover of planted perennial species, percentage of cover of non-planted, weedy species, and percentage of cover of bare ground. We ran frequentist linear models that investigated *C*. *fasciculata* abundance as a relationship of the interaction between herbivore treatment and the various cover types.

## Results

### Establishment kernel

**Fig 2:**
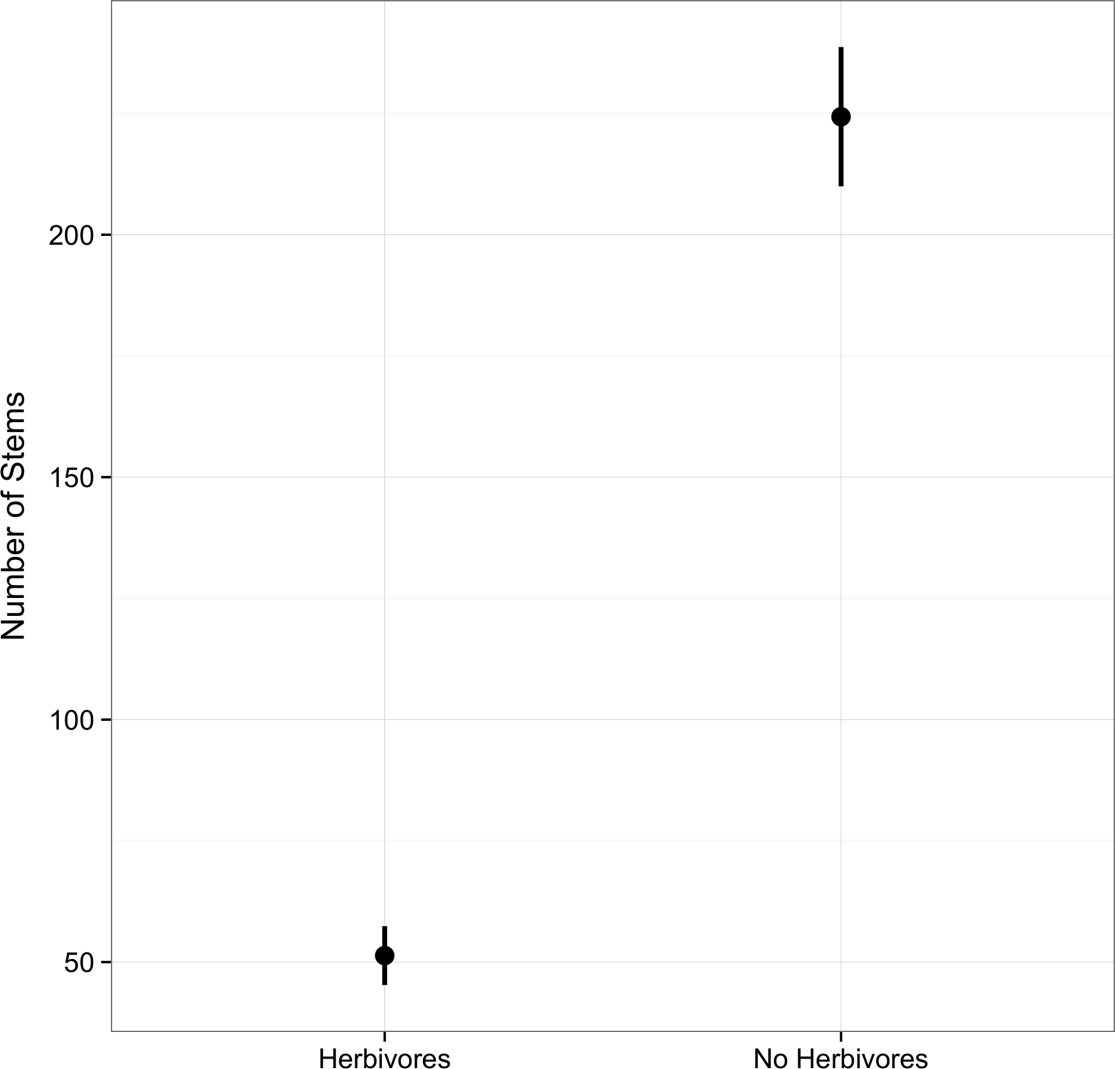
Mammalian herbivore effects on population growth. Herbivore presence significantly decreases the number of stems of *C*. *fasciculata* by 77%. Points represent model estimates with standard error bars.

**Fig 3:**
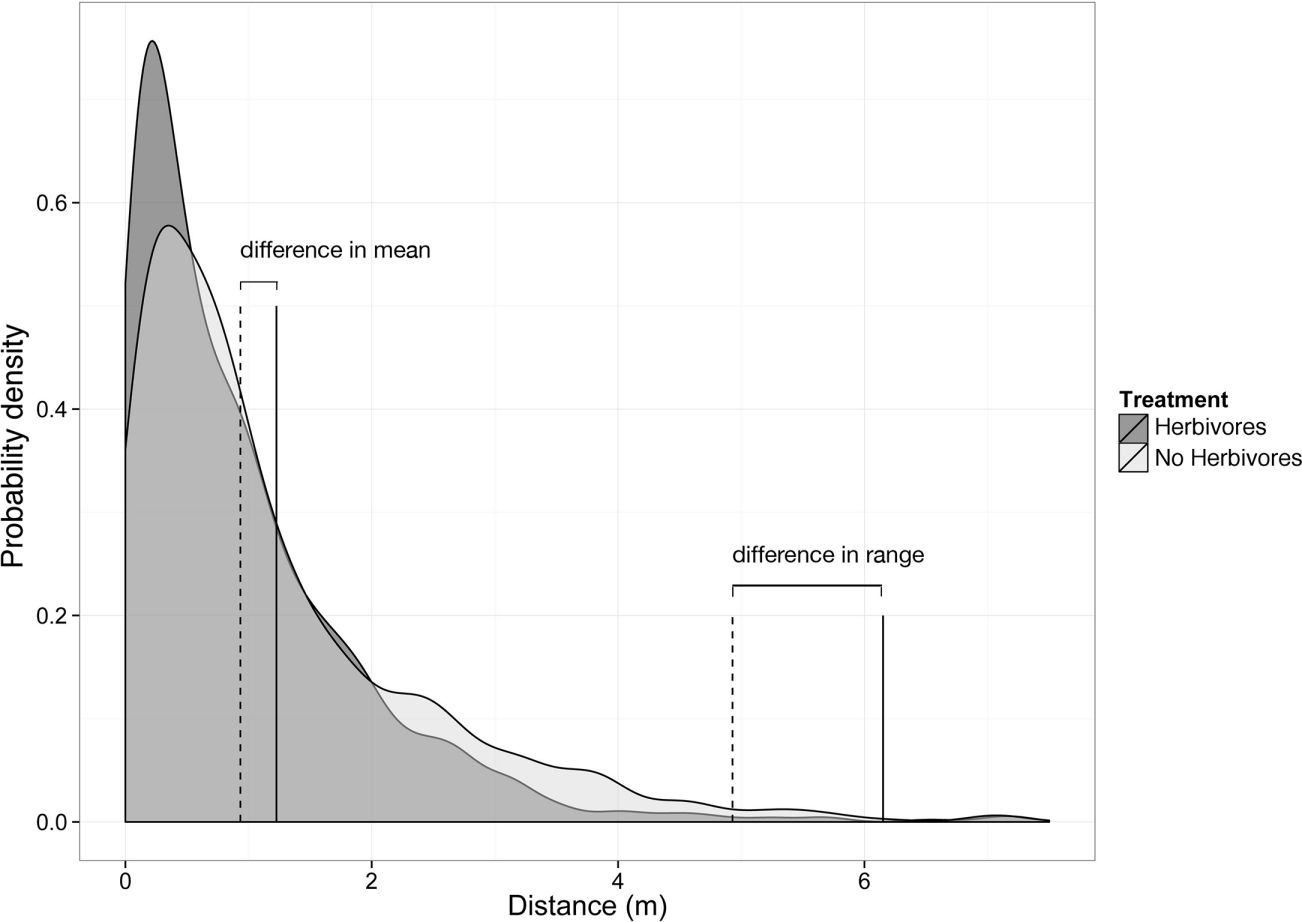
Simulated establishment kernels from model parameters. Mammalian herbivores reduced mean and range establishment distance of *C*. *fasciculata* by ~0.25m, and ~1.2m respectively. The dark gray kernel and hatched lines are from the plots where herbivores were present, light gray kernels and solid lines from where herbivores were absent.

When considering the body of the establishment kernel, herbivores significantly decreased the population growth of *C*. *fasciculata* by 77% (ANOVA; F = 41.01; p = 0.003; Fig 2), and decreased the population-level mean value for the establishment kernel (μ) by ~0.25 m (Fig 3). The approx. 0.90 quantile of the credible set in the herbivore treatment equals the approx. 0.10 quantile of the credible set in the non-herbivore treatment, indicating the distributions of μ′s per treatment overlap by ~10%. Similarly, herbivores affected the tail of the establishment kernel by decreased the range by ~1.2 m (herbivore effect on establishment tail ranged from 5%: .26m, to 95%: 2.25m; Fig 3).

### Competitive environment

**Fig 4:**
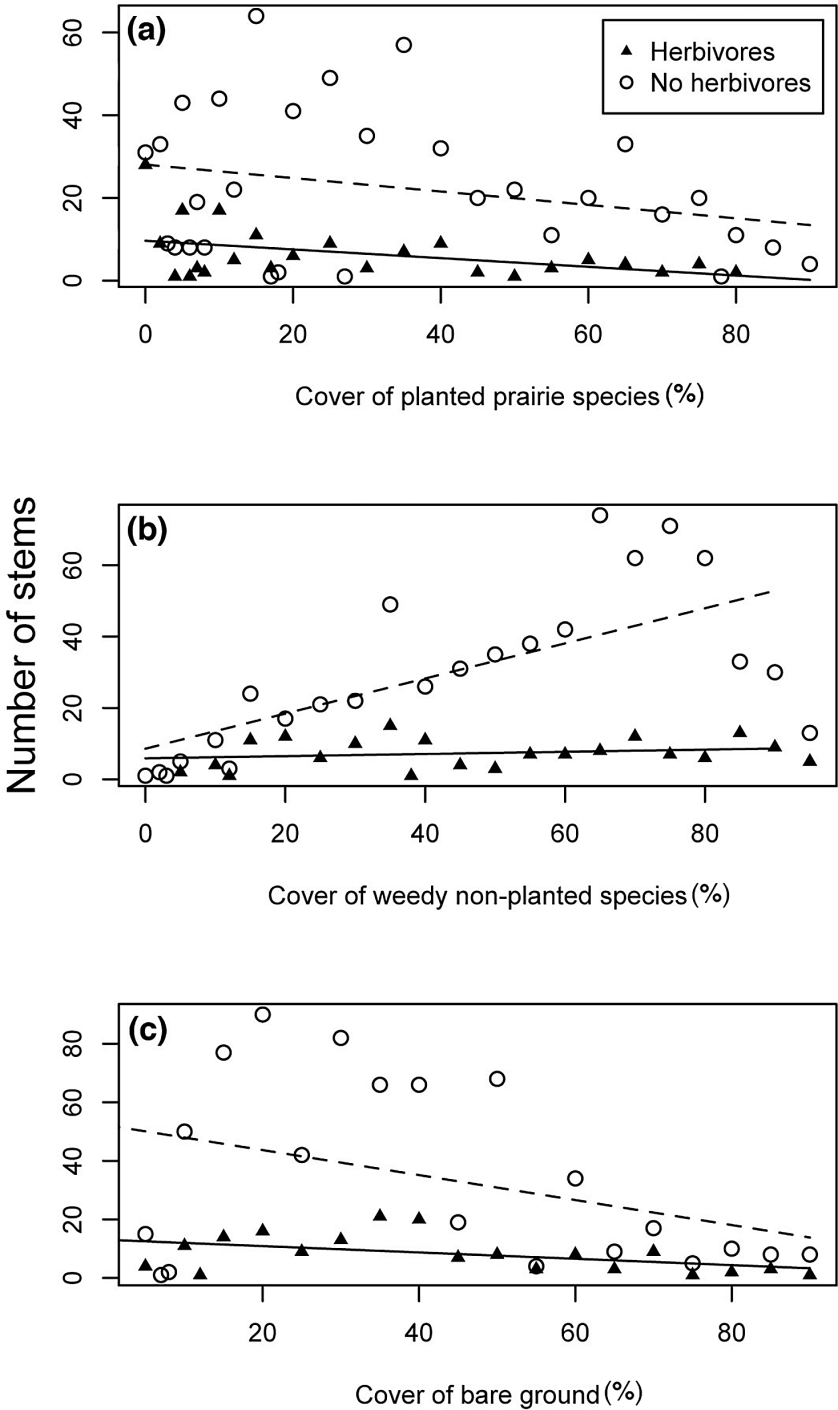
The community context of *C*. *fasciculata* establishment. The community context altered the establishment of *C*. *fasciculata* as measured by population size, or number of stems, and this context was altered by mammalian herbivore presence. Herbivores, but not native planted cover decreased *C*. *fasciculata* population size (a). Weedy, non-planted cover increased *C*. *fasciculata* establishment in the absence of herbivores, but there was no relationship when herbivores are present, indicating an interaction between herbivore presence and cover of planted species (b). Increased bare ground, and herbivore presence decreased *C*. *fasciculata* establishment, but there was no interaction between the two (c).

The competitive environment around each *C*. *fasciculata* stem had an effect on its establishment, and in some cases the slope of this relationship was modified by herbivores. The cover of native prairie species had a marginal, but not significant, negative effect on *C*. *fasciculata* establishment (F = 3.149; p = 0.082), and herbivores tended to decrease the number of stems established overall (F = 20.15; p <0.0001), with no significant interaction. This indicates that herbivores influenced establishment, but did not change the linear relationship between establishment and native prairie cover (Fig 4). In contrast, *C*. *fasciculata* establishment was positively affected by the cover of weedy species (F = 15.72; p<0.001). Herbivores again decreased establishment (F = 38.58; p<0.0001), but this time there was a statistically significant interaction, showing herbivores altered the slope of this relationship (F = 12.28; p = 0.0011; Fig 4). Finally, there was a negative relationship between bare ground cover and the establishment of *C*. *fasciculata* (F = 5.43; p = 0.025), and herbivores again decreased establishment (F = 13.31; p<0.001), with no significant interaction (Fig 4).

## Discussion

Our results showed that mammalian herbivores strongly impacted the population growth, establishment kernel, and establishment context of *C*. *fasciculata* in an assembling tallgrass prairie (Figs 2 and 3). This experiment tested how native grassland species can invade novel territory in the presence and absence of herbivores. The two key factors in determining a species' invasion rate into a novel community is its population growth, as well as its dispersal kernel, which is explicitly linked to the establishment kernel [19]. While we did not directly measure the invasion rate of *C*. *fasciculata*, the large decrease (77%) in population growth and moderate decrease in mean (.25m) and range (1.2m) of the establishment kernel suggests potential differences in invasion rate in the presence or absence of herbivores. We found evidence that *C*. *fasciculata* mean establishment distance was displaced toward the source population when herbivores were present. This indicates that herbivores could slow some native species as they attempt to shift their ranges in response to climate change. As many native plant species face interactions with these same herbivores and are likely similarly palatable, these dynamics are likely to generalize to other plant-herbivore systems. However, it is important to keep in mind that plant-herbivore interactions can be complex, and can involve non-mammalian herbivores as well. For example, insect herbivores can have significant influence on plant dynamics, and are likely to influence establishment kernels by altering population size, either directly through decreasing viable seed output via seed predation [e.g.: 37,38], indirectly through above and belowground herbivory that leads to decreased seed production [39], or through altering the community context of the individuals that are moving [40].

While our work relates to the Janzen-Connell hypothesis [41,42], that predicts specific relationships between adult plants and their established offspring in relation to natural enemies, our work does not directly test the Janzen-Connell hypothesis. Most studies directly test this hypothesis by placing seeds, seedlings or adult plants in known locations and allow natural enemies to remove individual plants (e.g.: removal from seed caches, consumption of planted adults, etc) and determine the pattern of removal [17,43]. Our study is quite different, in that we allow herbivores to determine the pattern of spatial establishment by affecting the source population only, thus providing new insight into movement ecology. While it is true that herbivores could consume established seedlings after they move, we did not control where these seedlings moved to, and thus we are not directly asking questions related to the Janzen-Connell hypothesis.

Our observed decrease in mean establishment distance of *C*. *fasciculata* could be due to herbivore alterations to adult plant height, which is an important trait related to plant movement [13]. Grasslands differ from forest communities, where most distance-dependent work was conducted [17,44], in that herbivores not only consume of seeds and seedlings, but they can also consume significant portions of adult plants. It is unlikely that herbivores significantly influence the height of seed release in most forests once trees have reached a height above the browse line. In grasslands, however, we suggest that significant alterations to the adult source population via partial-consumption could, in part, be influencing mean establishment distance, if this consumption alters the height from which *C*. *fasciculata* seeds explosively disperse. *C*. *fasciculata* adult plants can be partially consumed, where the top portion of the plant is removed by herbivores, but the bottom portion still produces flowers and seeds. Empirically validated dispersal models suggest that shorter plants disperse seeds shorter distances [45,46]. The change in height caused by herbivores may have contributed to the decrease in mean establishment distance we found in the herbivory treatment (Fig 3).

Interestingly, while the change in mean distance between herbivore treatments was relatively small, herbivores had a much larger effect on the range, or tail, of the distribution. The larger difference in establishment range with herbivore treatment is likely because the NB distribution is positively skewed. Based on simulations from our derived *μ* and *α* parameters, the range cutoff (i.e., 1% of the establishment kernel [21,47]) was 1.2m farther in the plots without herbivores (Fig 3). This distance difference could have a significant impact on invasion rate of *C*. *fasciculata* over generations, as the range of 6.2m without herbivores is significantly farther than the dispersal distances reported in the literature for this species. Fenster [48] measured the mean establishment distance of *C*. *fasciculata* at 0.65m, with a maximum distance of 3.0m in open habitat. This may be due in part to the fact that these seeds are establishing in a matrix of prairie that is also in the process of establishing, as more niches are available. While our establishment tail may not represent true regional, long distance dispersal because our transects are only 10m long, we are getting a good estimate of the local-scale tail of establishment. Based on simulations of potential dispersal distances of *C*. *fasciculata* from an empirically validated dispersal model (i.e.: the WALD model [45,49]), the tallest individuals would disperse 95% of seeds ~14m, and 99% of seeds ~26m under average wind conditions during dispersal months.

Herbivores also played a role in determining how well *C*. *fasciculata* individuals established in various types of plant communities (Fig 4), which could influence future coexistence and diversity. While not significant, as the cover of other native species increased, the number of establishing stems of *C*. *fasciculata* tended to decrease. Herbivores decreased overall establishment, but did not change the slope of the relationship between *C*. *fasciculata* establishment and cover of native prairie species. Based on these results, it is likely that *C*. *fasciculata* is a poorer competitor for nutrients and light when competing with other native perennials locally. This follows competition-colonization theory, that species that are strong colonizers (e.g., annuals) will be less dominant than strong competitors (e.g., perennials) and will rely on moving to open habitat for establishment [50], which reinforces the importance of dispersal for *C*. *fasciculata* as herbivores decrease opportunities for finding safe microsites. In contrast, as the cover of weedy, non-planted species increased, the number of *C*. *fasciculata* stems tended to increase, indicating that *C*. *fasciculata* is a better competitor than other, smaller seeded, weedy species in the restoration. Interestingly, herbivores appear to remove the competitive advantage *C*. *fasciculata* has in weedy environments. This could occur through an increase in conspicuousness, and therefore preference, when *C*. *fasciculata* is in the presence of higher weed densities [51,52]. However, if the herbivores strongly prefer weedy species to native perennials and feed or cache relatively indiscriminately in these areas, *C*. *fasciculata* could be a casualty to overall higher consumption rates by herbivores [53]. This result was not likely due to increased seed consumption by mice, as they were not excluded from plots, and population sizes were similar across treatments.

Here, we show that herbivores decrease both population growth and the mean of the establishment kernel of a native annual (*C*. *fasciculata*) in an establishing tallgrass prairie. Previous work on small-mammal communities found that these animals influenced the establishment, richness, and diversity of grassland restorations [28]. The novelty of our work is in showing that herbivores influence the spatial establishment context of seeds. These results have implications for the invasion rate of this native species, and indicate that herbivore presence could decrease connectivity in grassland habitats. Restored and reconstructed grassland systems tend to have lower overall species diversity as compared to remnants [54], and lose this diversity over time [55]. Herbivore influence on the growth and movement of native species in these systems could play a role in this diversity loss.

## Supporting Information

S1 Code R code for analyses and figures. (TEX)

S1 Data Population growth and establishment distance data. (CSV)

S1 Fig Fit of various distributions to the establishment distance data. (TIFF)
