# Supplementary figures and images for: Mammalian Herbivores Alter the Population Growth and Spatial Establishment of an Early-Establishing Grassland Species
Source: PLoS One. 2016 Feb 5;11(2):e0147715. doi: 10.1371/journal.pone.0147715 (PMC4743957; doi:10.1371/journal.pone.0147715)

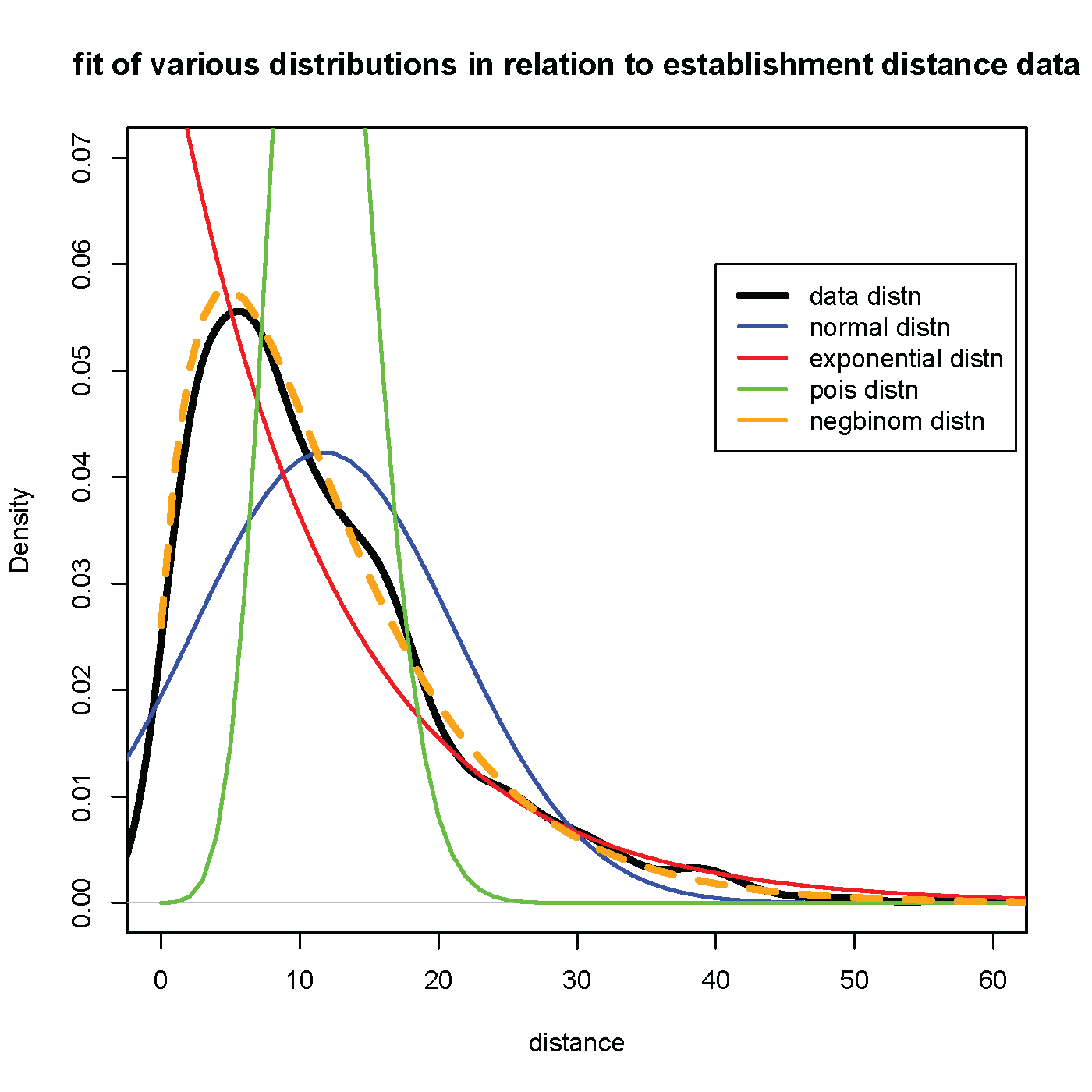

Supplement: S1 Fig — (TIFF) [file pone.0147715.s003.tiff]
